# Health-related quality of life of female patients with congenital adrenal hyperplasia in Malaysia

**DOI:** 10.1186/s12955-020-01515-9

**Published:** 2020-08-01

**Authors:** Ani Amelia Zainuddin, Sonia Regina Grover, Nur Azurah Abdul Ghani, Loo Ling Wu, Rahmah Rasat, Mohd. Rizal Abdul Manaf, Khadijah Shamsuddin, Zaleha Abdullah Mahdy

**Affiliations:** 1 Department of Obstetrics & Gynaecology, Faculty of Medicine, National University of Malaysia (UKM), Jalan Yaacob Latif, Bandar Tun Razak, Cheras, 56000 Kuala Lumpur, Malaysia; 2 Faculty of Medicine, Dentistry and Health Sciences, Royal Children’s Hospital, Melbourne, Australia; 3 Department of Paediatrics, University of Melbourne, Melbourne, Australia; 4 Department of Paediatrics, Faculty of Medicine, National University of Malaysia (UKM), Kuala Lumpur, Malaysia; 5 Department of Public Health, Faculty of Medicine, National University of Malaysia (UKM), Kuala Lumpur, Malaysia

**Keywords:** Health-related quality of life, Congenital adrenal hyperplasia, Disorders of sex development

## Abstract

**Background:**

This study investigates the health-related quality of life (HRQOL) of female patients with congenital adrenal hyperplasia (CAH) in Malaysia. The objectives were to attain socio-demographic and medical data on these Malaysian females with CAH and establish their health-related quality of life (HRQOL) in comparison to age matched diabetic controls.

**Methods:**

A cross-sectional study was conducted over 6 months in the two main tertiary centres for CAH patients in Malaysia. Participants including 59 female-raised CAH patients (mean age ± SD = 16.3 ± 4.2 years, range 10–28 years) compared to 57 age-matched female diabetic patients (mean age ± SD = 16.5 ± 3.4 years, range 10–26 years). Socio-demographic and medical profiles was obtained through semi-structured interviews. HRQOL of participants were evaluated utilising validated, Malay translated questionnaires which were age appropriate: Pediatric Quality of Life Inventory (PedsQL v4.0) scales for Child (8–12) and Adolescent (13–18) and Medical Outcome Survey 36-item Short Form version. These were then compared to the diabetic controls.

**Results:**

The CAH participants consisted of children (ages 10–12 years, *n* = 12), adolescents (ages 13–17 years, *n* = 29) and adults (≥ 18 years, *n* = 18). The majority were Malays (64.4%) and had salt-wasting CAH (67.8%). There were no significant differences between the total mean score of the HRQOL of the combined children and adolescents CAH group (total mean score ± SD = 81.6 ± 17.9, 95% CI = 75.6–87.6) when compared to age-matched diabetic patients (total mean score ± SD = 80.8 ± 11.0, 95% CI = 77.0–84.5, *P* = 0.81, effect size = 0.05); no significant difference between the adult CAH and diabetic controls in the physical [median score (IQR) CAH vs diabetics; 49.3 (11.4) vs. 50.2 (6.1), *P* = 0.60, effect size = 0.09] and the mental composite scores [median score (IQR) CAH vs. diabetics; 47.8 (14.1) vs. 50.0 (10.8), *P* = 0.93, effect size = 0.01].

**Conclusions:**

The HRQOL of the Malaysian CAH cohort were comparable to the diabetic controls.

## Background

Congenital adrenal hyperplasia (CAH) consists of an enzyme deficiency, in over 90% of cases due to 21-hydroxylase deficiency (21-OHD) [1]. It is associated with a spectrum of phenotypes, from the classical forms of potentially life-threatening, salt-wasting (SW) and the less severe, simple-virilizing (SV) usually apparent at birth in affected females as genital virilisation. The non-classical form (NC-CAH) manifests as increasing virilisation before or at puberty, without the genital ambiguity [2]. This condition is potentially life- threatening, and requires life long steroid hormone replacement and monitoring, thus regular hospital visits are necessary for both males and females. CAH has significant additional implications for females due to excessive androgen exposure and consequent virilisation. There is genital virilisation present at birth in the classic forms of CAH with potential issues relating to gender of rearing and the impact of any feminising surgery that may be undertaken. As a consequence, there are a range of psychological, psychosexual and fertility issues confronting these women [3–7]. In developing nations, these patients face additional challenges of poverty, ignorance, poor basic medical knowledge, lack of psychological services and poor access to healthcare [3, 8–11].

Malaysia is a rapidly developing country in Southeast Asia, with a multi-ethnic Asian population. As a conservative family-oriented society, young women who are studying or young women who are not married tend to remain at home with their families. Pre-marital sex is still considered taboo. Psychological issues are not addressed openly in Malaysian communities as mental health is still associated with stigma. Access to psychological care is also limited due to the lack of clinical psychologists. Cultural taboos regarding discussing sexuality-related issues mean that open discussion regarding these topics is limited.

Health-related quality of life (HRQOL) is an increasingly used outcome measure used for chronic conditions [12], thus assessment in this cohort of patients with a complex, chronic condition is important. Comparison with a control group who also share many attributes, offers the opportunity to understand differences that are inherent to the conditions. Thus comparison to patients with diabetes, a condition which also requires regular outpatient attendances, daily medications, the potential of adverse effects due to medications, the risk of decompensation during inter-current illnesses, the potential of life-threatening crises, physical effects from their medical disorder, potential adverse impacts on family life, schooling as well as social life. Thus, if differences in the health-related quality of life outcomes between these groups were found, it is more likely attributed to their specific medical disorder and not merely the presence of a chronic medical condition.

This paper reports on the care and health related quality of life (HRQOL) of genetically female (46, XX) patients with 21-hydroxylase deficiency (21-OHD) CAH raised female in Malaysia as compared to age-matched diabetic patients. There is very little published data on this cohort in Malaysia or other developing Asian countries and it is unclear whether the problems faced or the HRQOL may be different to those with other chronic medical conditions or to the outcomes experienced in western countries.

## Methods

Participants in this cross-sectional study were genetically female patients with 21-OHD CAH, aged > 10 years and raised female. Databases from two centres in Malaysia [University Kebangsaan Malaysia (National University of Malaysia) Medical Centre (UKMMC) and University Malaya Medical Centre (UMMC)] as well as the Paediatric Adolescent Gynaecology (PAG) unit in UKMMC were searched to identify potential participants. Those who attended follow up within the six-month study period and fulfilled the selection criteria were invited to participate. Controls were age-matched patients recruited from diabetic clinics in UKM. The first part describes the socio-demographic and medical profiles of the study cohort, while the second part compares health-related quality of life (HRQOL) of the CAH patients with the control group of female patients with diabetes.

Potential participants were informed about the study by their usual clinicians if they attended clinics between March 2012 to September 2012. The researchers further explained the study and gained informed consent from those who agreed to participate. For participants aged < 18 years old, the consent was obtained from their parent or legal guardian in addition to the verbal consent from the young person.

Recruitment and completion of questionnaires were undertaken at the time of clinic appointment to avoid extra cost and inconvenience to the patients and families. Data collection included measurement of height and weight. A semi-structured interview was undertaken to obtain data on socio-demographic and medical profiles, and then participants completed a validated Bahasa Malaysia versions of HRQOL questionnaires. Permission for the use of these questionnaires was obtained from the relevant institutions. Medical records were also reviewed.

The study was approved by research committees of the respective medical centres (UKM Medical Research and Ethics Committee, Approval Reference FF-318-2011 and UMMC Medical Ethics Committee, Approval Reference 859.11).

### Evaluating the health-related quality of life (HRQOL)

Age-appropriate questionnaires to assess the health-related quality of life (HRQOL) were used, with participants divided into age groups to match this. For the 10–12(age group I) and the 13–18 years old (adolescents, age group II), the Pediatric Quality of Life Inventory generic core (Version 4.0) (PedsQL v4.0) scales for Child (8–12) and Adolescent or Teens (13–18), respectively were used. The PedsQL 4.0 has 23 items including (1) Physical Functioning (8 items), (2) Emotional Functioning (5 items), (3) Social Functioning (5 items) and (4) School Functioning (5 items). Higher scores indicate better HRQOL [13]. Parents who accompanied their children completed the parent-proxy reports.

The reliability and validity of the PedsQL have been assessed in both physically healthy populations and patients with acute and chronic health conditions. The Bahasa Malaysia version of the questionnaires, which was previously utilised in Malaysian studies, were found to have a Cronbach alpha > 0.70 for each of the domains and an overall Cronbach alpha value of 0.86, thus was used in this study [14].

For participants ≥ 18 years (age group III), the Medical Outcome Survey 36-item Short Form version 2 (SF 36v2) in Bahasa Malaysia and English version were utilised. This generic indicator of health status has been used in population surveys and to assess outcomes of various health conditions. Data from a Malaysian general population cohort of > 3000 men and women [15] provides useful reference baselines for Malaysian studies. Domain scores range from 0 to 100 with higher values denoting better subjective health status [16].

### Statistical analyses

Data were analysed using SPSS version 20.0. Descriptive analyses were carried out for the CAH respondents included mean ± standard deviation (SD), median (Md), interquartile ranges (IQR), ranges for continuous variables. Frequencies and percentages were used for categorical variables. Normality of the distribution of data was determined by Kolmogorov-Smirnov tests and by reviewing histograms. Comparison of specific outcomes of the CAH group with controls, for continuous variables, utilised independent t-tests for normally distributed data and Mann Whitney U tests for non-normally distributed data. For categorical variables, Pearson Chi square test for association was used with *p* ≤ 0.05 to indicate statistical significance.

Effect size (ES) or “strength of association” cannot be assessed purely from *p*-values [17]. The effect size, r, was calculated for the data from the health-related questionnaires PedsQL 4.0 Generic Core Scales and the SF-36 and Cohen’s criteria of 0.1 = small effect, 0.3 = medium effect and 0.5 = large effect [18] were used to interpret these results.

## Results

### Profile of the CAH participants

Of the 61 potential participants identified, 59 consented to participate. Distance travelled by participants was up to 400 km. The socio-demographic details of the CAH participants are described in Table 1. The mean age was 16.3 ± 4.24 years (range 10–28 years), with 12, 29 and 18 participants distributed into the age groups of I, II, III, respectively.

**Table 1:** Sociodemographic profiles of the CAH participants

Variables	CAH participants							
	Age group I 10–12 years (*n* = 12)		Age group II 13–17 years (*n* = 29)		Age group III ≥ 18 years (*n* = 18)		Total *N* = 59	
	f	%	f	%	f	%	f	%
**Ethnicity**								
Malay	9	75.0	19	65.5	10	55.6	38	64.4
Chinese	0	–	7	24.1	7	38.9	14	23.7
Indian	3	25.0	3	10.3	1	5.6	7	11.9
**Religion**								
Muslim	9	75.0	19	65.5	10	55.6	38	64.4
Others	3	25.0	10	34.5	8	44.4	21	35.6
**Present education**								
Primary	12	100.0	6	20.7	0	–	18	30.5
Secondary	0	–	20	68.9	7	38.9	27	45.8
Tertiary	0	–	2	6.9	7	38.9	9	15.3
Not completed secondary education	0	–	1	3.4	4	22.2	5	8.5
**Occupation**								
Student	12	100.0	28	96.6	4	22.2	44	74.6
Employed	0	–	1	3.4	11	61.1	12	20.3
Non-employed	0	–	0	–	3	16.7	3	5.1
**Average family income/monthly (RM)**								
< 2000	6	50.0	10	34.5	7	38.9	23	39.0
≥ 2000	6	50.0	19	65.5	11	61.1	36	61.0

In the study group, five participants did not complete secondary school education. Three of these had recognised learning disabilities. As the majority of the participants were in the younger age groups, most were still students.

Only one CAH participant was married. Only two were sexually active; one, a 28 year old married participant (NC-CAH); the other, a 24 years old postgraduate student (SV-CAH).

Most CAH participants came from families with average monthly incomes above the poverty line for Malaysia (at the time of data collection this was Malaysian Ringgit (RM) 2000.00/household/month, equivalent to $USD666.70).

Table 2 shows the medical profiles, including height and prevalence of overweight/obesity of CAH participants. The majority were SW-CAH and only one of the non-classical variants. The SW-CAH variants were diagnosed early in life (from newborn up to 6 months). For the SV-CAH participants, despite having ambiguous genitalia, the age range for the diagnosis was newborn period to 13 years. The sole NC-CAH participant was diagnosed at age 11 years.

**Table 2:** Medical profiles and heights of the CAH participants

Variables	CAH participants							
	Age group I 10–12 years (*n* = 12)		Age group II 13–17 years (*n* = 29)		Age group III ≥ 18 years (*n* = 18)		Total *N* = 59	
	f	%	f	%	f	%	f	%
**CAH variants**								
SW-CAH	7	58.3	18	62.1	15	83.3	40	67.8
SV-CAH	5	41.7	11	37.9	2	11.1	18	30.5
NC-CAH	0	–	0	–	1	5.6	1	1.7
**Daily corticosteroid replacement**								
Prednisolone	4	33.3	17	58.6	13	72.2	34	57.6
Hydrocortisone	8	66.7	12	41.4	5	27.8	25	42.2
**On mineralocorticoids**	10	83.3	23	79.3	17	94.4	50	84.7
**Hypertensive**	0	0	3	10.3	3	16.7	6	10.2
**Participants’ perceived compliance to medications**								
Excellent	3	25.0	11	37.9	6	33.3	30	33.9
Intermediate	8	66.7	13	44.8	8	44.4	29	49.2
Poor	1	8.3	5	17.2	4	22.2	10	16.9
**Other co-morbidities**	1	8.3	9	31.0	7	38.9	17	28.8
**Had precocious puberty**	6	50.0	12	41.4	3	16.7	21	35.6
**Learning disability**	2	16.7	6	20.7	2	11.1	10	16.9
**Affected sibling (s)**	5	41.7	15	51.7	8	44.4	28	47.5
**Height (cm), Mean (SD)**	142.7 (9.0)		151.0 (6.8)		149.0 (5.6)			
**Obese or overweight**	5	41.6	10	34.4	6	33.4	21	35.6

Medication use is shown in Table 2, with mineralocorticoids used in the majority and prednisolone increasingly used with increasing age. Almost a third of the CAH participants had other co-morbidities including epilepsy, hypertension, polycystic ovarian syndrome, congenital hypothyroidism and asthma. Additional medications used by CAH participants included Amlodipine, Decapeptyl, Metformin, combined oral contraceptives to regulate their menstrual cycles and L-Thyroxine.

All participants with classical CAH had ambiguous genitalia, with all but four having undergone feminizing genitoplasty in childhood.

Table 2 depicts the CAH participants’ perceived compliance with their glucocorticoid and mineralocorticoid medications. A third report excellent compliance, however, in adulthood, more respondents admitted to poor compliance, as compared to their two younger counterparts.

Data of height for each age group were normally distributed. The mean body mass index (BMI) for the CAH participants was 23.0 ± 5.3 kg/m^2^ (range: 15.2–35.8 kg/m^2^). For those < 18 years (children and teens), BMI values were plotted on the Centers for Disease Control and Prevention (CDC) BMI-for-age growth charts for girls to obtain a percentile ranking and then categorized accordingly, into underweight, healthy weight, overweight or obese. For those participants > 18 years, BMI values were classified according to the World Health Organization’s recommendations where a BMI of 25–29.9 kg/m^2^ = overweight or pre-obese and ≥ 30 kg/m = obese (19). More than a third (33.4–41.6%) in each age group of CAH participants were overweight/obese.

### Comparison of CAH participants with diabetic controls

Table 3 compares the socio-demographic profiles of the 57 age-matched diabetic patients (control group) with the CAH participants. Mann Whitney U test revealed that there was no significant difference in the ages of the CAH group (Md = 16.0 years) and the control group (Md = 16.0), *U* = 1584, *z* = − 0.540, *P* = 0.59, *r* = 0.05. The only significant difference between the study group and the control group was ethnicity, with the majority of the control group being non-Malays in contrast to the CAH participants. Controls were recruited from the diabetic clinics of UKMMC, situated in an urban area, where there are higher numbers of non-Malays compared to Malays. Additionally, there are many diabetic clinics throughout Malaysia, whereas there are only two main tertiary centres in Malaysia, where the majority of CAH patients attend.

**Table 3:** Comparing the socio-demographic profiles of the CAH group with the control group

Variables	CAH group (*n* = 59)		Control group (*n* = 61)		*P*-value
	f	%	F	%	
**Ethnicity**					
Malay	38	64.4	24	42.1	0.03 ^α^ - S
Others	21	35.6	33	57.9	
**Religion**					
Muslim	38	64.4	28	49.1	0.14 ^α^
Others	21	35.6	29	50.9	
**Education level**					
Primary	18	30.5	10	17.5	
Secondary	32	54.2	33	57.9	0.19 ^β^
Tertiary	9	15.3	14	24.6	
**Occupation**					
Student	44	74.6	45	78.9	0.74 ^α^
Non-student	15	25.4	12	21.1	
**Average family income/month (RM)**					
< 2000	23	39.0	19	33.3	0.66 ^α^
> 2000	36	61.0	38	66.7	

^α^ Yates Continuity Correction tests, significance level < 0.05

^β^ Pearson Chi-Square test

S- significant at *p* < 0.05

While five (8.5%) CAH participants did not complete their secondary education, all the controls did so. No controls were married. The two groups are thus comparable.

### Comparing the health-related quality of life between the CAH children and teenagers and their age-matched diabetic controls

Twenty of the 10–12-year-old participants (12 CAH participants and eight controls) completed the child self-report with 20 of their parents answering the parent-proxy reports. Of the 53 adolescents, who completed the Teens self-report, 27 were CAH participants and 28 controls. As items and scoring in each of the PedsQL™ 4.0 are identical, the Means and SDs were calculated for combined children and adolescent CAH participants (*n* = 39) and the controls (*n* = 36) and for their parents (*n* = 39 for the study group and *n* = 21 for the controls) to increase the sample sizes (Table 4).

**Table 4:** Scale descriptives for PedsQL™ 4.0 Generic Core Scales Teen Self-Reports and Parent Proxy- Reports

Scale	CAH participants			Control group participants			*P*-value	Effect size r
	n	Median	IQR	n	Median	IQR		
**Teen Self-Reports**								
Physical	27	93.8	25.00	28	87.5	21.10	0.19	0.18
Emotional	26	85.0	35.00	28	65.0	28.75	0.03*	0.30
Social	26	95.0	20.00	28	95.0	20.0	0.80	0.03
School	26	75.0	28.75	28	70.0	20.00	0.25	0.16
Psychosocial	24	83.3	24.17	28	77.5	19.58	0.09	0.23
Total score	24	89.3	18.88	28	82.19	19.17	0.09	0.24
**Parent Proxy-Reports**								
Physical	27	81.3	56.25	13	68.8	39.06	0.78	0.05
Emotional	27	75.0	35.00	13	90.0	32.50	0.16	0.22
Social	27	90.0	40.00	13	90.0	20.00	0.15	0.16
School	26	72.5	50.00	13	75.0	22.50	1.00	0
Psychosocial	26	72.5	37.50	13	80.0	16.67	0.36	0.15
Total score	26	78.9	45.91	13	74.4	27.97	0.53	0.10

Scale descriptives for PedsQL™ 4.0 Generic Core Scales Teen Self-Reports and Parent Proxy- Reports and comparative analyses between the CAH and control groups using Mann Whitney U tests

*Significant at *p* < 0.05

Each CAH participant had been accompanied by their parent(s). However, only 21 of the control group participants was accompanied by parents(s). Hence there were less parent-proxy reports in the control group.

Except for the physical and social functioning scales, the CAH participants had higher mean scores than control group respondents; however, these differences were not significant with small effect sizes. Interestingly, the parents of the CAH participants reported lower mean scores of the scales compared to their children who were also lower when compared to the parents of the control group, with moderate effect sizes and in the opposite direction. There was a significant difference in the social functioning scale between the parents of the CAH group and the controls.

### Comparing HRQOL between the adult CAH participants and controls

A total of 17/18 (94.4%) CAH and 20/21 (95.2%) control participants completed the SF36v2. Mann Whitney U tests were used to compare the results as the scores were not normally distributed (Table 5).

**Table 5:** Comparing the scale scores of SF36 between the adult CAH participants and adult control participants

Variables	CAH group (*n* = 17)		Control group (*n* = 20)		*P*-value	Effect size *r*
	Median	IQR	Median	IQR		
**Domains SF-36**						
Physical Functioning (PF)	90.0	15.00	90.0	17.50	0.651	0.07
Role-Physical (RP)	100.0	37.50	100.0	25.00	0.490	0.11
Bodily Pain (BP)	84.0	32.50	79.0	43.75	0.512	0.11
General Health (GH)	62.0	27.50	57.0	25.25	0.714	0.06
Vitality (VT)	70.0	25.00	70.0	28.75	0.759	0.05
Social Functioning (SF)	87.5	31.25	75.0	25.00	0.147	0.24
Role-Emotional (RE)	66.7	66.67	100.0	33.30	0.204	0.21
Mental Health (MH)	80.0	28.00	74.0	16.00	0.691	0.06
Physical Composite Score (PCS)	49.3	11.42	50.2	6.13	0.604	0.09
Mental Composite Score (MCS)	47.8	14.11	50.0	10.83	0.927	0.01

The comparison of the Domain/scale scores for SF36 between the adult CAH participants (*n* = 17) and the adult control participants (*n* = 20), using Mann Whitney U tests with effect sizes

Similar to their younger counterparts, there were no significant differences in all scales of the HRQOL. The effect sizes were within the small range (0.01–0.24). The median scores for the physical and mental composite scores for the CAH cases were lower compared to the controls but not significant.

## Discussion

The ethnicity of the 59 CAH participants in this study corresponded to the distribution of ethnic groups in Malaysia. According to Department of Statistics of Malaysia (2018), there were 69.1% Bumiputera which includes Malays, 23.0% Chinese and 3.9% Indians [19].

The majority of the participants were < 18 years old, with a mean age of 16.3 ± 4.24 years. The first paediatric endocrine clinic in Malaysia was started in UKMMC in 1990 and hydrocortisone was first available in Malaysia in 1993 (as disclosed by Professor Wu L. L., the first paediatric endocrinologist in Malaysia). It is likely that prior to the 1990s, babies with classical CAH did not survive in Malaysia. The numbers of neonates and children that have died with undiagnosed CAH in Malaysia over the last few decades are not known. This report can thus only present data on the survivors, and of those, only those who have continued specialist care. It is also not known how many of the CAH patients have defaulted follow-up, how many genetically female CAH patients have been raised male, or whether there were others that have assumed a male gender and not been identified.

Challenges with completing education amongst CAH patients has been reported in a recent Swedish study where women with CAH had completed primary education less often than controls [20]. Possible reasons given include learning disabilities, steroid treatments possibly affecting cognitive functions such as memory or difficulty in concentration, and psychological and social problems encountered by these patients at school [20]. These explanations corresponded to the reasons given by CAH participants in our study.

This study revealed that with increasing age, the CAH participants have an increasing rate of co-morbidities, rising rates of requiring other medications and reduced rates of perceived self-compliance to their daily medications. The finding of increased morbidities is in agreement with other studies on adult patients with CAH that have shown that they have increased co-morbidities which include metabolic abnormalities such as obesity, hypercholesterolaemia, insulin resistance, osteopenia and osteoporosis [21–23].

The mean height (SD) for the adult CAH participants was 5 cm shorter than the mean age-adjusted height of a Malaysian female of 154.0 cm (SE = 0.1 cm) according to the National Health and Morbidity Survey (NHMS) [24]. Loss of height have been extensively reported in CAH patients, with a recent systematic review and meta-analysis reporting that mean final height was − 1.38 SDS and mean corrected height was − 1.03 SDS (final height SDS – midparental height SDS) [25] thought to be as a result of hyperandrogenism and/or hypercortisolism [26]. In several studies, classical CAH patients appear to be trending towards better height achievement in younger (< 30 years) patients [25, 27, 28] presumably due to better CAH management. This trend is also observed in our study, and it may be attributed to the earlier diagnoses with treatment in the younger participants.

Problems with CAH patients of all phenotypes being overweight or obese has been reported by others with rates of one third or higher [21, 26]. This could be due to the effects of steroid treatments. According to the National Health and Morbidity Survey (NHMS), 26.5% of the adult Malaysian population was overweight [24]. Meanwhile, less than 33.4% of our adult CAH population were overweight/obese.

The reports on the HRQOL have been variable for women with CAH [3], ranging from better quality of life compared to control groups [29, 30], through to similar HRQOL [31, 32] to worse [21, 23]. Recently, there have been reports of quality of life studies in children and adolescents with CAH [33, 34].

In a study reporting no significant difference in HRQOL between the CAH patients and their control diabetic group, Kunhle et al. (1995) [32] explained that patients with chronic disease may develop coping strategies and cognitive appraisals that enable them to accept their life and view it as satisfying but remain impaired socially and sexually. The findings in our study, where both the CAH and control groups have reported similar HRQOL, may reflect both having adjusted to their respective chronic diseases [35].

The total mean score for CAH participants for PedsQL™4.0 was higher when compared to diabetic controls, although not significant. Reisch et al. [30] explained that the finding of better subjective HRQOL in CAH in general compared to the group with primary adrenal insufficiency (PAI), reflected that the differences of HRQOL perception could be due to a congenital versus acquired disease. PAI, compared their HRQOL before and after the onset of disease, whereas classical CAH patients have always lived with their disease [30]. This may be the case in our younger CAH participants as they were diagnosed either at birth or early in childhood, whereas the average age of diagnosis in the diabetic controls was at 8 years old. The argument does not hold for our older population, where many with SV-CAH had their diagnosis made in later childhood.

In comparison to a Brazilian study who used the same HRQOL instrument, our mean (SD) scores for each domain and total scores were comparatively higher [34]. The CAH participants in this Brazilian study also had significantly reduced scores compared to the healthy controls [34]. The causes of this are unclear.

Our study findings were more in agreement with the Dutch study, which also investigated different aspects of the functioning of children with CAH and their parents [33]. They found that children with CAH experience few negative effects, they participate well in school and leisure time, and the CAH adolescents gave a high rate for their level of functioning [33].

The mean scores for every scale of the PedsQL™4.0 for the parent-proxy reports were lower compared to the self-reports of the children and teenagers with CAH and parent-proxy reports of the diabetic controls. This suggests that the parents of the CAH participants have more negative perceptions of their children’s’ HRQOL compared to their children; likewise, they had more negative perceptions than the control parents. In contrast, the Dutch study [33] reported parent-reported QOL of their children was not impaired in any dimension. Differences between self and proxy report in subjective HRQOL have been described for other chronic diseases [36]. One explanation for some of the difference may lie with the secretive nature of the diagnosis of CAH in Malaysia. There has not been full disclosure of the disorder to the children, or they may not have a thorough understanding of CAH. Thus, the burden of the disorder lies almost entirely on the parents’ shoulders. Due to lack of psychological support for the parents, lack of educational materials in the national language which they can access and lack of a CAH support group, the cultural taboo and stigma associated with this condition, parents may be indicating their burden by these low scores in the parent-proxy reports. Compared to the diabetic families and potentially in comparison to other cultures, the capacity to openly discuss their diagnoses, the virilisation at birth, concerns regarding genital surgery and future fertility may all be impacting on parental anxieties. Future qualitative studies would help to clarify the reasons why parents in our study have more negative perceptions of their children’s’ HRQOL comparatively.

Several other studies have interestingly noted that impaired QOL is more likely in adults than in children, often associated with severity of CAH [31, 32, 37]. A possible reason may be that co-morbidity is more frequent in the adult population related to increasingly poor compliance among adults. Sanches et al. (2012) reported that 17% of their adolescents reported non-compliance with CAH-related medication compared to only 6% recognised by their parents [33]. Our study also shows that the prevalence of poor compliance increases as age increases.

When we compared our adult CAH population with those of Arlt et al. (2010) [21] and Nermoen et al. (2010) [23] who also used the SF36 to assess HRQOL, we found that our CAH adults had the lowest median score for RE (role emotional). This section asks how much emotional problems affect their work and other activities. Our population had a higher score on all other scales when compared to these two studies. Thus, our CAH participants subjectively had better physical, mental and social health but were impaired in their emotional health. The low emotional scores may be due to the lack of psychological support available in Malaysian hospitals, and which the patients and their families are reluctant to receive anyway. The lack of openness in Malaysian society to discuss this diagnosis may also contribute to the lower emotional scores. In terms of physical health, our adult population is much younger compared to the mean ages of the other two studies [21, 23]. Thus, the rates of co-morbidities are probably lower. In terms of better social health, all of the CAH adult except for the married NC-CAH participants were living with their families and thus, they were not socially isolated. As the majority of our CAH adult population are not yet sexually active and many do not expect yet to get married due to their young age, the average age of marriage for a Malaysian woman being 25.3 years [38], psychosexual and fertility concerns are not yet prevalent in our study population.

When we compared our study results to the normal Malaysian female population aged 18–29 years (similar ages to our study), our CAH adult participants’ scores were higher in domains of role physical, bodily pain, vitality, social functioning and mental health, had the same scores for physical functioning and had lower scores for general health and role emotional domains compared to the normal Malaysian young women [15]. It was not suprising that the normal Malaysian population had higher scores for general health but suprising that our participants scored higher subjectively in the other domains. This may be due to the relative sample sizes as the sample size in Azman’s study was 445 and in this study, 17 CAH participants completed the SF-36.

The limitation of our study is the small sample size due to the rare incident of this disease. Thus, we were unable to apply detailed statistical models. A repeat study on the same cohort may give different results, i.e., lower quality of life as the patients have grown up and get married. Some of them may have problems with sexual intercourse and conceiving or not be able to get married.

## Conclusion

The findings show that at present, the HRQOL of the Malaysian CAH female participants is satisfactory. However, reassessment, when this cohort is older, would be appropriate as HRQOL may worsen due to social isolation, sexual and infertility issues, which may become more apparent with increasing age. Thus, the medical team managing this population needs to incorporate mental health experts as well as gynaecologists and implement thoughtfully planned and coordinated transitional care from paediatric to adult medical care.

## Data Availability

All data generated or analysed during this study are included in this published article [and its supplementary information files].
